# Dynamic predictive accuracy of electrocardiographic biomarkers of sudden cardiac death within a survival framework: the Atherosclerosis Risk in Communities (ARIC) study

**DOI:** 10.1186/s12872-019-1234-9

**Published:** 2019-11-14

**Authors:** Erick A. Perez-Alday, Aron Bender, David German, Srini V. Mukundan, Christopher Hamilton, Jason A. Thomas, Yin Li-Pershing, Larisa G. Tereshchenko

**Affiliations:** 1 Knight Cardiovascular Institute, Department of Medicine, Oregon Health & Science University School of Medicine, 3181 SW Sam Jackson Park Rd; UHN62, Portland, OR 97239 USA; 2 Department of Biomedical Informatics, Emory University, Atlanta, GA USA; 3 UCLA Cardiac Arrhythmia Center, University of California Los Angeles, Los Angeles, CA USA; 4 Rush University, Chicago, IL USA; 5 Rosalind Franklin University of Medicine and Science, North Chicago, IL USA; 6 University of Washington, Seattle, WA USA; 7 Cardiovascular Division, Department of Medicine, Johns Hopkins University School of Medicine, Baltimore, MD USA

**Keywords:** Electrocardiography, Sudden cardiac death, Vectorcardiography, Dynamic prediction, Global electrical heterogeneity

## Abstract

**Background:**

The risk of sudden cardiac death (SCD) is known to be dynamic. However, the accuracy of a dynamic SCD prediction is unknown. We aimed to measure the dynamic predictive accuracy of ECG biomarkers of SCD and competing non-sudden cardiac death (non-SCD).

**Methods:**

Atherosclerosis Risk In Community study participants with analyzable ECGs in sinus rhythm were included (*n* = 15,716; 55% female, 73% white, age 54.2 ± 5.8 y). ECGs of 5 follow-up visits were analyzed. Global electrical heterogeneity and traditional ECG metrics (heart rate, QRS, QTc) were measured. Adjudicated SCD was the primary outcome; non-SCD was the competing outcome. Time-dependent area under the receiver operating characteristic curve (ROC(t) AUC) analysis was performed to assess the prediction accuracy of a continuous biomarker in a period of 3,6,9 months, and 1,2,3,5,10, and 15 years using a survival analysis framework. Reclassification improvement as compared to clinical risk factors (age, sex, race, diabetes, hypertension, coronary heart disease, stroke) was measured.

**Results:**

Over a median 24.4 y follow-up, there were 577 SCDs (incidence 1.76 (95%CI 1.63–1.91)/1000 person-years), and 829 non-SCDs [2.55 (95%CI 2.37–2.71)]. No ECG biomarkers predicted SCD within 3 months after ECG recording. Within 6 months, spatial ventricular gradient (SVG) elevation predicted SCD (AUC 0.706; 95%CI 0.526–0.886), but not a non-SCD (AUC 0.527; 95%CI 0.303–0.75). SVG elevation more accurately predicted SCD if the ECG was recorded 6 months before SCD (AUC 0.706; 95%CI 0.526–0.886) than 2 years before SCD (AUC 0.608; 95%CI 0.515–0.701). Within the first 3 months after ECG recording, only SVG azimuth improved reclassification of the risk beyond clinical risk factors: 18% of SCD events were reclassified from low or intermediate risk to a high-risk category. QRS-T angle was the strongest long-term predictor of SCD (AUC 0.710; 95%CI 0.668–0.753 for ECG recorded within 10 years before SCD).

**Conclusion:**

Short-term and long-term predictive accuracy of ECG biomarkers of SCD differed, reflecting differences in transient vs. persistent SCD substrates. The dynamic predictive accuracy of ECG biomarkers should be considered for competing SCD risk scores. The distinction between markers predicting short-term and long-term events may represent the difference between markers heralding SCD (triggers or transient substrates) versus markers identifying persistent substrate.

## Background

Sudden cardiac death (SCD) is a major contributor to cardiovascular mortality, accounting for 40–50% of the years of potential life lost from all cardiovascular diseases (CVD) [1, 2]. In the United States (US), more than 350,000 emergency medical services-assessed out-of-hospital sudden cardiac arrests occur annually [1]. There remains a lack of reliable, dynamic predictors of SCD [3]. An electrocardiogram (ECG) can characterize the presence and properties of the electrophysiological substrate of SCD. Our group recently showed that global electrical heterogeneity (GEH), as measured by five metrics [spatial QRS-T angle, spatial ventricular gradient (SVG) azimuth, elevation, and magnitude, and sum absolute QRST integral (SAI QRST)] is independently (after comprehensive adjustment for time-updated CVD events and their risk factors) associated with SCD, representing an underlying substrate of SCD [4]. The subsequent discovery of 10 genetic loci, associated with GEH at a genome-wide significance level, confirmed the presence of several underlying mechanisms behind the GEH ECG phenotype [5]. We also developed a competing risk score of SCD and showed that the addition of GEH measures to clinical risk factors significantly improves the reclassification of SCD risk [4]. The risk of SCD is known to be dynamic. However, current risk models predict SCD using baseline risk factors measured at a single point in time. Therefore, evaluating the accuracy of a dynamic prediction is, therefore, necessary to better understand the temporal relationship between substrate and events. The goal of this study was to investigate the dynamic predictive accuracy of GEH and traditional ECG biomarkers of SCD within a survival framework in comparison with competing non-sudden cardiac death (non-SCD) in the Atherosclerosis Risk in Community (ARIC) study participants.

## Methods

### Study population

The ARIC study is an ongoing prospective cohort study evaluating risk factors, progression, and outcomes of atherosclerosis in 15,792 participants (45% male, 74% white) enrolled in four US communities in 1987–1989. The ARIC study protocol and design have been previously described [6]. We excluded ARIC participants with absent or poor-quality ECGs due to noise, artifacts, or missing leads (*n* = 24), atrial fibrillation (AF) (*n* = 36), and ventricular pacing (*n* = 16) at the baseline study visit. Only participants in normal sinus rhythm were included in this study (*n* = 15,716). If AF or ventricular pacing were diagnosed at any time during follow-up, such participants were included for a period until AF or ventricular pacing were diagnosed on 12-lead ECG. Prevalent CVD was defined as the presence of at least one baseline prevalent condition: coronary heart disease (CHD), heart failure (HF), stroke, peripheral artery disease (PAD), atrioventricular (AV) block II-III, atrial or ventricular pacing, or Wolff-Parkinson-White ECG phenotype.

### Clinical characteristics of participants

Prevalent CHD was defined as a history of myocardial infarction (MI), or coronary revascularization via coronary artery bypass surgery or percutaneous coronary intervention. Prevalent MI was defined as a self-reported history of MI and/or ECG evidence of MI as defined by the Minnesota code [7]. Prevalent AF was defined as either a self-reported and validated history of AF or a diagnosis of AF on the baseline ECG. Prevalent HF was defined as self-reported use of HF medication or evidence of symptomatic HF as defined by stage 3 of the Gothenburg criteria [8], which required the presence of specific cardiac and pulmonary symptoms in addition to medical treatment of HF. Prevalent stroke in ARIC was defined by a stroke and transient ischemic attack diagnostic algorithm, as previously described [9]. PAD was defined as the ankle-brachial index ≤0.90. Details of ankle-brachial index measurement in the ARIC study have been previously described [10].

### Definition of a primary outcome: sudden cardiac death

A follow-up of ARIC participants was previously reported [11]. SCD was defined as a sudden pulseless condition in a previously stable individual without evidence of a non-cardiac cause of cardiac arrest if the cardiac arrest occurred out of the hospital or in the emergency room. To identify cases of SCD in ARIC, cases of fatal CHD that occurred by December 31, 2012 were reviewed and adjudicated by a committee of physicians in two phases, as previously described [12]. CHD deaths occurring on or before December 31, 2001 were adjudicated in the first phase. CHD deaths occurring between January 1, 2002 and December 31, 2012 were adjudicated in the second phase. Available data from death certificates, informant interviews, physician questionnaires, coroner reports, prior medical history, and hospital discharge summaries were reviewed, in addition to circumstances surrounding the event. Each event was adjudicated independently by two physicians. In cases of disagreement, a third reviewer independently reviewed the event to provide final classification. Definite, probable, or possible SCD was included in this study as a primary outcome: the strength of available evidence determined this stratification. Definite SCD included cases of witnessed SCD with definite evidence such as an available rhythm strip of life-threatening cardiac arrhythmia, or primary emergency medical services impression of cardiac arrest. Probable SCD was defined as SCD with uncertainty either due to concomitant clinical conditions that can muddle the exact cause of demise, or limited information to adjudicate an event. Possible SCDs were adjudicated only in the second phase of reviews. The strength of evidence for probable SCD was greater than the strength of evidence for possible SCD. Possible SCD included cases of death that were unwitnessed but had specified SCD on a death certificate and did not document another cause of death. Participants were censored at the time of loss to follow-up, incident AF or ventricular pacing on 12-lead ECG, or death if the cause of death was any other than SCD.

### Competing mortality outcome: non-sudden cardiac death

Cases of fatal CHD were adjudicated by the ARIC Morbidity and Mortality Classification Committee, as previously described [11]. Fatal CHD that did not meet the criteria of SCD comprised the non-SCD outcome.

### 12 lead ECG recording

12-lead ECG was recorded according to the ARIC study protocol and manual (version 1.0; August 1987). The method and procedure for 12-lead ECG recording, as described in the ARIC manual, are outlined below. During the baseline examination, a standard supine 12-lead ECG was recorded after a 12-h fast followed by a light snack and at least 1 h after smoking or ingestion of caffeine. The standard electrocardiograph for the ARIC study was the MAC PC by Marquette Electronics, Inc. A 12-lead resting ECG was obtained consisting of 10 s of each of the leads (I, II, III, aVR, aVL, aVF, Vl-V6) simultaneously recorded. In an effort to enable longitudinal comparisons of ECGs, ARIC investigators developed and implemented a uniform procedure for electrode placement and skin preparation. The participant, stripped to the waist, was instructed to lie on the recording bed with arms relaxed at the sides. The individual was asked to avoid movements that may cause errors in marking the electrode locations. For optimal electrode/skin interface, the electrodes were placed on the skin at least 2–3 min before recording the ECG. A pen was used to mark the six chest electrode positions. The chest was wiped with a sterile alcohol prep. Left leg electrode was placed on the medial surface of the left ankle. Right leg electrode was placed on the medial surface of the right ankle. Left arm electrode was placed on the medial surface of the left wrist. Right arm electrode was placed on the medial surface of the right wrist. Electrode V1 was located in the 4th intercostal space at the right sternal border, immediately to the right of the sternum. Electrode V2 was located in the 4th intercostal space, immediately to the left of the sternal border. Next, E-point was located by finding the 5th intercostal space, and following it horizontally to the midsternal line. Location of V6 electrode was found using the chest square. V6 was located at the same level as the E point in the midaxillary line (straight down from the center of the armpit). If breast tissue was over the V6 area, V6 was placed on top of the breast. No attempt was made to move the breast. Electrode V4 was located using a flexible ruler, as a midway between E and V6. Electrode V3 was located using a flexible ruler, midway between V2 and V4. Using a flexible ruler, electrode V5 was located midway between the locations of V4 and V6. After placing the electrodes on the skin, the participant’s information was input to the MAC PC. It was required that electrodes must be on the skin for at least 3 min before taking the ECG. During the ECG recording, special attention was paid to the quality of recording. Quality control and technical troubleshooting procedures were in place to minimize errors (lead switch), noise, and artifacts. Recorded 12-lead ECGs were originally saved in the memory of the ECG machine and transmitted to a MUSE database (GE Marquette, Milwaukee, WI) within the Halifax ECG Computing Center via the phone line. Later, the MUSE database was transferred from the Halifax ECG Computing Center to the Epidemiological Cardiology Research Center (EPICARE, Wake Forest University, NC), and then to the Tereshchenko laboratory at Oregon Health & Science University.

### Electrocardiogram analyses

**Fig. 1:**
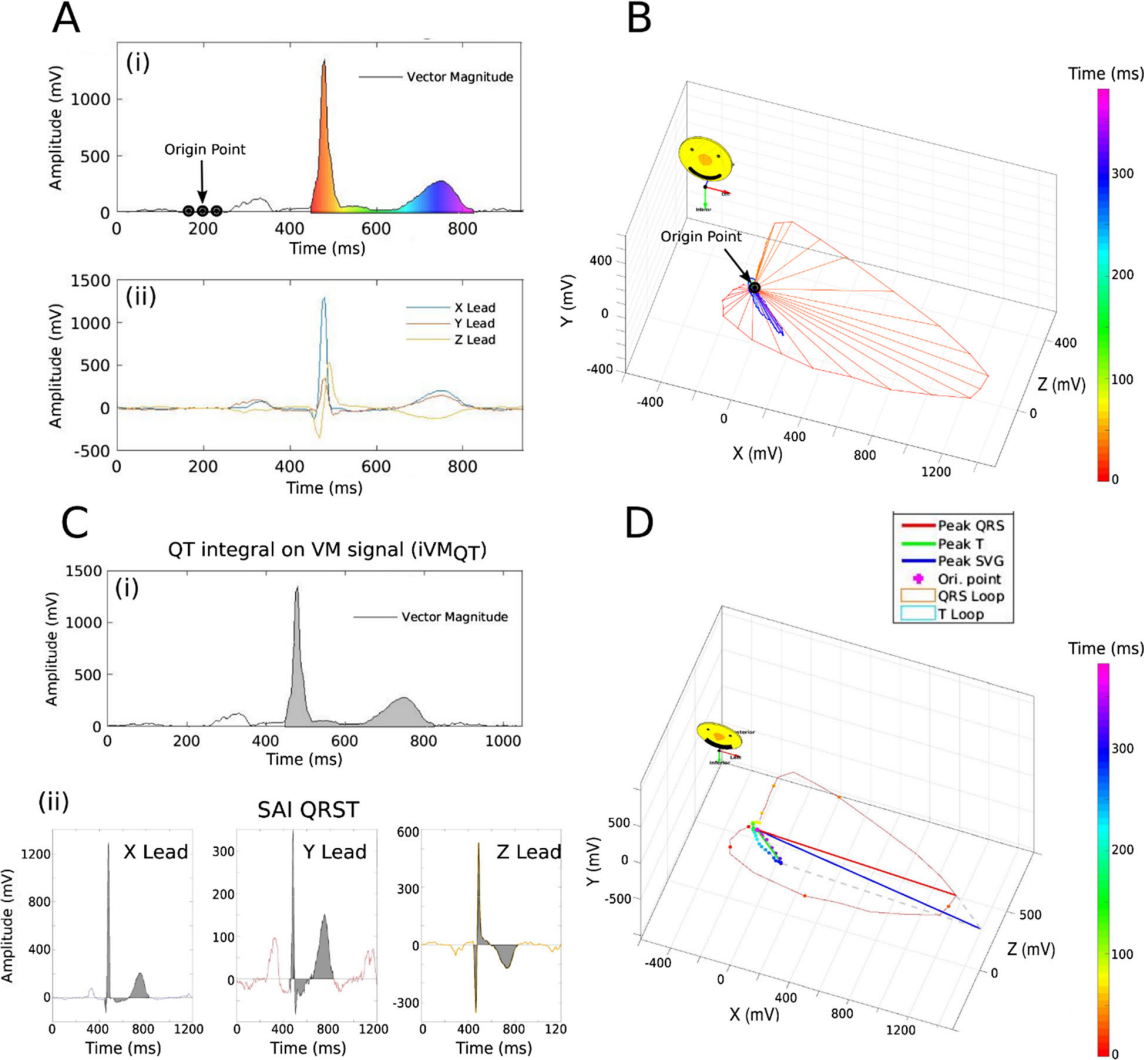
GEH measurement example of peak and area vectors, and vector magnitude (VM). **a** (i) VM plotted over time, and (ii) corresponding X, Y, and Z leads. **b** VM plotted in a three-dimensional space. Color-coded progression from QRS onset (red) to the end of T (purple) is shown. **c** Measurement of (i) QT integral on VM, and (ii) SAI QRST. **d**. Measurement of peak SVG vector magnitude, azimuth, and elevation

ECG data from all five follow-up visits were analyzed. Traditional ECG intervals were reported by the 12 SL algorithm using Magellan ECG Research Workstation V2 (GE Marquette Electronics, Milwaukee, WI). QT interval was corrected for heart rate according to Bazett’s formula. We analyzed raw, digital, 10-s, 12-lead ECGs (sampling rate of 500 Hz and amplitude resolution of 1 μV). Origin and conduction path of each cardiac beat was adjudicated by the team of physicians (DG, AB, SVM, LGT), and each beat was manually labeled by investigators (CH, JAT) for subsequent automated analyses. A representative normal sinus median beat was constructed. For the development of a normal sinus median beat, sinus beats before and after premature ventricular complexes, and noisy or distorted beats were excluded. In this study, only the normal sinus median beat was used for our analysis. GEH was measured as previously described, [4, 13] in a time-coherent median beat with the identified origin of the heart vector [14]. We have provided the open-source software code at Physionet (https://physionet.org/physiotools/geh/). In addition to previously reported “mean” GEH measures, [4] in this study we measured the spatial peak vectors (Fig. 1) [13–15]. First, we transformed the 12-lead ECG into an orthogonal XYZ ECG, using Kors transformation [16]. Next, we constructed a time-coherent median beat, and detected the origin of the heart vector using our novel approach, as recently described [14]. Then, we performed calculations of GEH metrics using the following equations.

### Spatial QRS-T angles

Spatial peak QRS-T angle was calculated as the 3-dimensional angle between the spatial peak QRS vector and the spatial peak T vector:

A.1 $$ Spatial\ peak\  QRS-T\  angle=\operatorname{arccos}\left(\frac{\overrightarrow{QRSpeak}\bullet \overrightarrow{Tpeak}}{\left| QRSpeak\right|\left| Tpeak\right|}\right) $$

Spatial area QRS-T angle was calculated as the 3-dimensional angle between the spatial area QRS vector and the spatial area T vector:

A.2 $$ Spatial\ area\  QRS-T\  angle=\operatorname{arccos}\left(\frac{\overrightarrow{QRSm} ean\bullet \overrightarrow{Tmean}}{\left| QRSmean\right|\left| Tmean\right|}\right) $$

Spatial ventricular gradient vectors:

Magnitude and direction of spatial area (Wilson’s) and peak SVG vectors were measured.

A.3 $$ \overrightarrow{SVG}V=\overrightarrow{QRSpeak}+\overrightarrow{Tpeak} $$

A.4 $$ Spatial\ Peak\  SVG\  Azimuth=\arctan \left(\frac{SVGV_Z\  dt}{SVGV_X\  dt}\right)\kern2em $$

A.5 $$ Spatial\ Peak\  SVG\  Elevation=\arctan \left(\frac{SVGV_X\  dt}{SVGV_Y\  dt}\right) $$

A.6 $$ SpatialPeakSVGMagnitude=\sqrt{SVG{V_X}^2+ SVG{V_Y}^2+ SVG{V_Z}^2} $$

A.7 $$ Spatial\ Area\  SVG\  Azimuth=\arctan \left(\frac{\int_{QRS- onset}^{T- offset}{V}_Z(t) dt}{\int_{QRS- onset}^{T- offset}{V}_X(t) dt}\right)\kern2em $$

A.8 $$ Spatial\ Area\  SVG\  Elevation=\arctan \left(\frac{\int_{QRS- onset}^{T- offset}{V}_X(t) dt}{\int_{QRS- onset}^{T- offset}{V}_Y(t) dt}\right) $$

Wilson’s (area) SVG magnitude was also calculated:

A.9 $$ \left| SVG\right|=\sqrt{{\left({\int}_{QBeg}^{TEnd}{V}_x(t) dt\right)}^2+{\left({\int}_{QBeg}^{TEnd}{V}_y(t) dt\right)}^2+{\left({\int}_{QBeg}^{TEnd}{V}_z(t) dt\right)}^2} $$

### Statistical analysis

Time-dependent area under the receiver operating characteristic curve (ROC(t)AUC) analysis was performed to assess the predictive accuracy of a continuous biomarker in a period of 3, 6, 9 months, and 1,2,3,5,10, and 15 years, using an unadjusted survival analysis framework approach [17, 18]. We used the nearest neighbor estimator, which allows the censoring to depend on the marker and is therefore realistic. The percentage of observations included in each neighborhood was defined by the eq. 0.25* $\Big(\sqrt[3]{n}$), where *n* is the number of observations. All available five visits’ ECG data were included in time-dependent AUC analysis [4]. To satisfy the requirement for ROC analysis, and to perform internal validation of study findings, we divided the dataset into five unique (non-overlapping) partitions. In each partition, a study participant was presented not more than once, with a unique time to event defined as time from ECG recording to the time of outcome (or censoring). If a participant had five ECGs recorded at five study visits, each ECG contributed to a different partition. Those participants who had less than 5 visits/ECGs were randomly distributed across 5 partitions. Bootstrapping with 500 replications was performed to determine a 95% confidence interval (CI) of ROC(t) AUC in each partition, separately. Then, ROC(t) AUC point estimates, and lower and upper boundaries of 95%CI were averaged across 5 partitions, then presented as a final summary result. To assess the statistical power of ROC(t) AUC analysis at each time period, we compared observed 95% CI width with expected 95% CI width. Expected 95% CI width was calculated for observed sample size and observed AUC values at each time period. We summarized clinical characteristics of study participants with an SCD outcome within the first 3 months, 3–6 months, 6 months-1 year, 1–2 years, 2–5 years, and more than 5 years after ECG recording in a longitudinal dataset, reporting between-participant standard deviation (SD) for continuous variables, and between-participant frequencies for categorical variables. We then assessed whether the addition of traditional ECG metrics (heart rate, QRS, QTc) and GEH metrics to our previously identified clinical risk factors of SCD [4] (age, sex, race, diabetes, hypertension, CHD, and stroke) resulted in better predictive accuracy for SCD and non-SCD within the first 3 months, 3–6 months, 6 months-1 year, 1–2 years, 2–5 years, and more than 5 years after ECG recording. We calculated absolute integrated discrimination improvement (IDI), and net reclassification improvement (NRI) using multivariate logistic regression [19, 20]. IDI estimates improvement in average sensitivity and specificity. We estimated category-free NRI and two-category NRI for events, defining the high-risk category as a ≥ 25% risk of SCD/non-SCD within the first 3 months, 3–6 months, and 6 months-1 year after ECG recording. The high-risk category for events occurring 1–2 years, 2–5 years, and more than 5 years after ECG recording was defined as ≥10% risk of SCD/non-SCD. Statistical analysis was performed using STATA MP 15.1 (StataCorp LP, College Station, TX, USA). A *P*-value of < 0.05 was considered significant. PASS 2019 Power Analysis and Sample Size Software (NCSS, LLC. Kaysville, Utah, USA) was used for the calculations of the expected 95% CI width.

## Results

### Study population

The clinical characteristics of the study population are shown in Table 1. Approximately half of the study participants were female, and 73% were white. Average traditional ECG parameters were normal. During the SCD adjudication, the inter-reviewer agreement was 83.2%, and agreement across phases was 92.5%. Over a median follow-up of 24.4 years, there were 577 SCDs (incidence 1.76 (95%CI 1.63–1.91) per 1000 person-years), and 829 non-SCDs (incidence 2.54 (95%CI 2.37–2.71) per 1000 person-years). SCD victims who died within the first 3 months after ECG recording were more likely to be CVD-free white males with fewer prevalent CVD risk factors. In contrast, SCD victims who died more than 5 years after ECG recording had nearly equal probabilities of being male or female, white or non-white (Table 1).

**Table 1:** Clinical characteristics of study population

SCD event within the following time interval after ECG recording:							
Characteristic	*n* = 15,716	1–90 days (*n* = 11; T = 1)	91–180 days (*n* = 16; T = 1)	181–365 d (*n* = 47; T = 1)	365–730 d (*n* = 84; T = 1)	731–1825 d (*n* = 495; T = 2.5)	> 5 years (*n* = 320; T = 1.1)
Age ± SD, years	54.2 ± 5.8	63 ± 5	58 ± 6	62 ± 6	60 ± 7	59 ± 6	62 ± 7
Female, n(%)	8680(55.2)	18%	31%	30%	29%	36%	42%
White, n(%)	11,431(72.7)	82%	50%	60%	60%	59%	56%
Diabetes, n(%)	1867(12.0)	40%	47%	39%	38%	39%	40%
Hypertension, n(%)	5475(35.0)	40%	60%	64%	68%	71%	68%
Anti-hypertensive drugs, n(%)	4803(30.6)	40%	53%	62%	70%	68%	66%
CHD, n(%)	756(4.8)	20%	33%	36%	39%	30%	25%
Heart failure, n(%)	732(4.7)	0	25%	15%	9%	10%	9%
Stroke, n(%)	269(1.7)	0	13%	7%	18%	10%	10%
Peripheral artery disease, n(%)	630(4.2)	0	0	14%	17%	9%	14%
Current smoking, n(%)	4107(26.2)	40%	47%	36%	39%	38%	29%
Body-mass-index±SD, kg/m^2^	27.7 ± 5.4	32.4 ± 5.8	29.2 ± 5.4	29.1 ± 6.7	28.8 ± 6.2	29.4 ± 5.9	30.4 ± 6.6
Total cholesterol±SD, mmol/L	5.6 ± 1.1	5.3 ± 0.8	5.0 ± 1.2	5.2 ± 1.2	5.2 ± 1.2	5.6 ± 1.0	5.3 ± 1.1
Triglycerides±SD, mmol/L	1.5 ± 1.0	2.3 ± 2.0	1.5 ± 0.7	1.7 ± 1.1	1.9 ± 1.5	1.7 ± 1.2	1.7 ± 1.2
Alcohol consumption±SD, g/wk	42.4 ± 97.0	11 ± 23	130 ± 390	28 ± 75	44 ± 94	46 ± 102	33 ± 76
Heart rate ± SD, bpm	66.3 ± 10.3	74 ± 13	68 ± 11	65 ± 10	69 ± 14	67 ± 10	66 ± 12
Corrected QT ± SD, ms	416.4 ± 19.7	428 ± 20	436 ± 44	429 ± 33	433 ± 35	423 ± 23	424 ± 22
QRS duration±SD, ms	92.3 ± 12.7	97 ± 16	99 ± 12	109 ± 24	105 ± 24	98 ± 18	98 ± 18
BBB/IVCD, n(%)	666(4.2)	9%	6%	19%	13%	12%	12%
LBBB, n(%)	111(0.7)	4.5%	3.1%	2.7%	3.0%	1.0%	1.2%

*T* average number of visits (ECGs) per participant

### Time-dependent AUC analyses of SCD and competing outcome

**Fig. 2:**
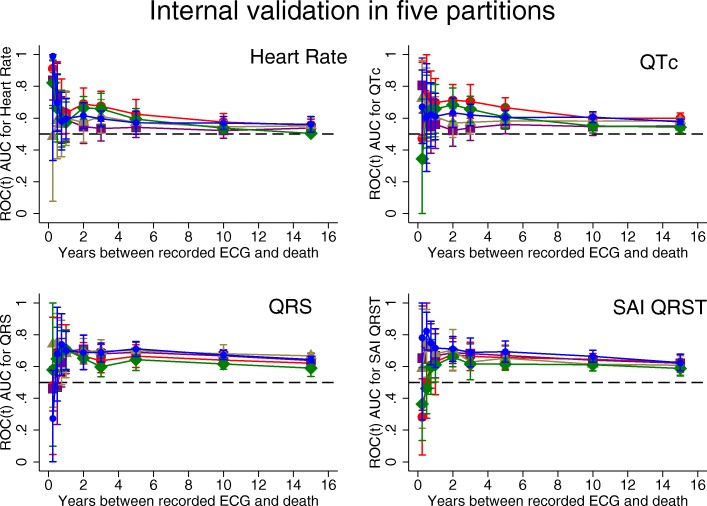
Internal validation of time-dependent AUC with 95% CI for prediction of SCD, for windows of prediction 3, 6, 9 months, 1, 2, 3, 5, 10, 15 years for heart rate, QTc, QRS duration, and SAI QRST in five partitions of the study dataset

**Fig. 3:**
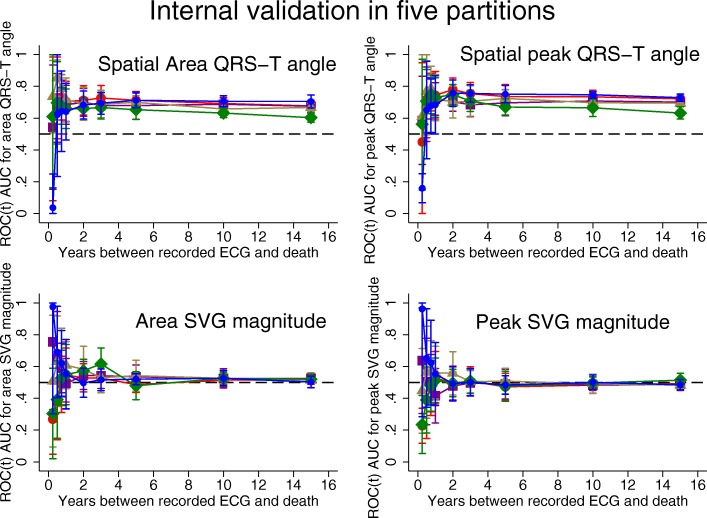
Internal validation of time-dependent AUC with 95% CI for prediction of SCD, for windows of prediction 3, 6, 9 months, 1, 2, 3, 5, 10, 15 years for spatial peak and area QRS-T angle and SVG magnitude in five partitions of the study dataset

**Fig. 4:**
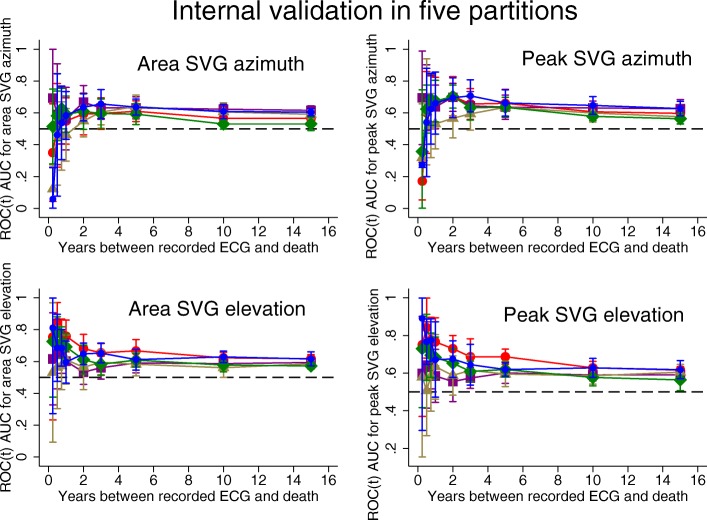
Internal validation of time-dependent AUC with 95% CI for prediction of SCD, for windows of prediction 3, 6, 9 months, 1, 2, 3, 5, 10, 15 years for peak and area SVG azimuth and elevation in five partitions of the study dataset

**Fig. 5:**
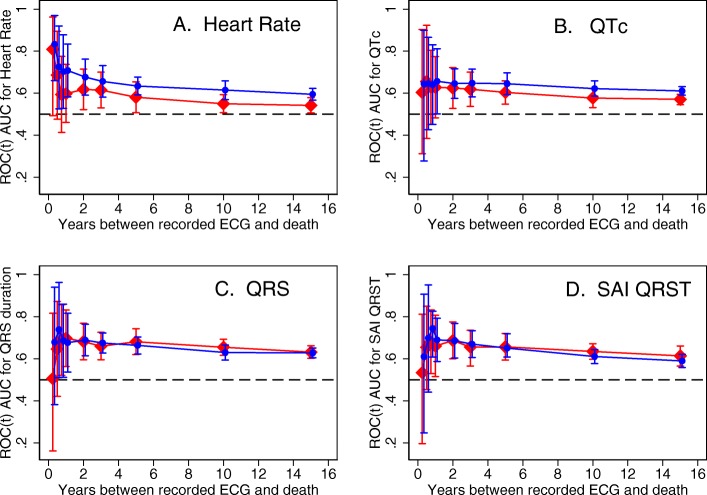
Time-dependent AUC with 95% CI for prediction of SCD (red diamonds), and non-SCD (blue circles) for windows of prediction 3, 6, 9 months, 1, 2, 3, 5, 10, 15 years for (**a**) heart rate, (**b**) QTc interval, (**c**) QRS duration, (**d**) SAI QRST measured at visits 1, 2, 3, 4, and 5

**Fig. 6:**
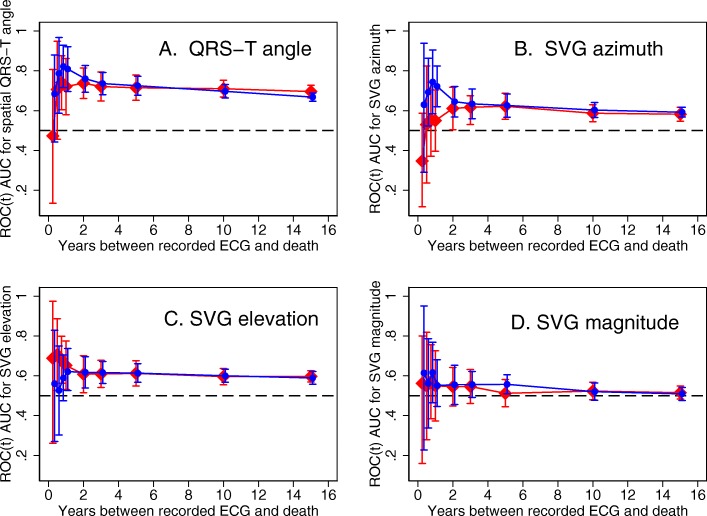
Time-dependent AUC with 95% CI for prediction of SCD (red diamonds), and non-SCD (blue circles) for windows of prediction 3, 6, 9 months, 1, 2, 3, 5, 10, 15 years for (**a**) spatial peak QRS-T angle, (**b**) area SVG azimuth, (**c**) area SVG elevation, (**d**) area SVG magnitude measured at visits 1, 2, 3, 4, and 5

Detailed results for all 5 partitions are provided in Additional file 1 Table S1 and Figs. 2, 3, 4. Considering the results of robust validation analysis, no ECG biomarkers predicted SCD within 3 months after ECG recording. Forward-directed SVG azimuth was significant predictor in some, but not all partitions: peak SVG azimuth partition #1 AUC 0.828 (95%CI 0.709–0.947), and partition #2 AUC 0.727 (95%CI 0.712–0.742). Predicted 95%CI width (Table 2) indicated insufficient statistical power for prediction of SCD within 3 months after ECG recording for most ECG biomarkers. At 6 months, SVG elevation was the only short-term statistically significant biomarker of SCD (area SVG elevation AUC 0.706; 95%CI 0.526–0.886), which specifically predicted SCD, but did not predict non-SCD (Fig. 6c). Figure 6 illustrates trends in variations in SVG vector direction (azimuth and elevation) predicting SCD versus non-SCD within 9 months – 1 year after ECG recording, when upward, and more likely forward-directed SVG vector predicted SCD, whereas leaning backward-directed SVG predicted non-SCD. In contrast, long-term (2 years and beyond) predictors of SCD and non-SCD had many similarities. Wide spatial QRS-T angle and backward [towards left ventricle (LV)] – directed SVG vector predicted both SCD and non-SCD. The most accurate prediction of SCD was provided by ECG biomarkers recorded within 2 years before the outcome (Figs. 5, 6). Most ECG biomarkers predicted both mortality outcomes, with few exceptions. SVG magnitude did not predict any outcome. At any time, heart rate and QTc were stronger predictors of non-SCD than SCD (Fig. 5). Neither heart rate nor QTc improved risk reclassification beyond traditional clinical risk factors of SCD (Table 3), whereas QRS duration improved reclassification of SCD risk for events occurring 2–5 years after ECG recording. Within the first 3 months after ECG recording, only SVG azimuth improved reclassification of the risk beyond traditional clinical risk factors (18% SCD events were reclassified from a low or intermediate-risk category to a high-risk category). All GEH metrics significantly improved reclassification of both SCD and non-SCD beyond clinical risk factors for events occurring at least 1 year after ECG recording (Table 3).

**Table 2:** Calculated/predicted and observed two-sided 95% confidence interval width for a range of observed AUC values

					*Calculated / predicted*			*Observed*		
*Time*	*N events*	*n non-events*	*ECG predictor*	*ROC AUC*	*95%CI width*	*Lower CI limit*	*Upper CI limit*	*ROC AUC*	*Lower CI limit*	*Upper CI limit*
*3 months*	*11*	*15,705*	*HR*	*0.809*	*0.311*	*0.654*	*0.964*	*0.809*	0.493	0.963
			*QTc*	0.604	*0.355*	*0.427*	*0.781*	0.604	0.312	0.903
			*QRS*	0.506	*0.343*	*0.335*	*0.677*	0.506	0.162	0.817
			*pQRST*	0.473	*0.335*	*0.306*	*0.640*	0.473	0.135	0.807
			*pSVGaz*	0.363	*0.293*	*0.216*	*0.510*	0.363	0.143	0.594
			*pSVGel*	0.71	*0.346*	*0.537*	*0.883*	0.71	0.306	0.98
			*pSVGmag*	0.502	*0.342*	*0.331*	*0.673*	0.502	0.189	0.687
			*aQRST*	0.492	*0.339*	*0.322*	*0.662*	0.492	0.181	0.826
			*aSVGaz*	0.347	*0.286*	*0.204*	*0.490*	0.347	0.117	0.587
			*aSVGel*	0.688	*0.350*	*0.513*	*0.863*	0.688	0.261	0.974
			*aSVGmag*	0.562	*0.352*	*0.386*	*0.738*	0.562	0.159	0.8
			*SAIQRST*	0.534	*0.346*	*0.360*	*0.708*	0.534	0.196	0.812
*6 months*	*16*	*15,700*	*HR*	0.686	*0.290*	*0.541*	*0.831*	0.686	0.477	0.895
			*QTc*	0.656	*0.293*	*0.509*	*0.803*	0.656	0.385	0.923
			*QRS*	0.647	*0.294*	*0.500*	*0.794*	0.647	0.421	0.873
			*pQRST*	0.706	*0.288*	*0.556*	*0.844*	0.706	0.456	0.949
			*pSVGaz*	0.613	*0.294*	*0.466*	*0.760*	0.613	0.356	0.871
			*pSVGel*	0.699	*0.288*	*0.556*	*0.844*	0.699	0.514	0.883
			*pSVGmag*	0.5	*0.283*	*0.358*	*0.642*	0.5	0.236	0.765
			*aQRST*	0.713	*0.286*	*0.570*	*0.856*	0.713	0.466	0.957
			*aSVGaz*	0.53	*0.289*	*0.390*	*0.678*	0.53	0.237	0.824
			*aSVGel*	0.706	*0.287*	*0.562*	*0.850*	0.706	0.526	0.886
			*aSVGmag*	0.549	*0.292*	*0.416*	*0.708*	0.549	0.279	0.819
			*SAIQRST*	0.655	*0.293*	*0.509*	*0.803*	0.655	0.454	0.85
*1 year*	*47*	*15,669*	*HR*	0.598	*0.172*	*0.512*	*0.684*	0.598	0.461	0.737
			*QTc*	0.628	*0.172*	*0.542*	*0.714*	0.628	0.482	0.774
			*QRS*	0.698	*0.168*	*0.614*	*0.784*	0.698	0.563	0.833
			*pQRST*	0.721	*0.166*	*0.638*	*0.805*	0.721	0.58	0.862
			*pSVGaz*	0.633	*0.172*	*0.547*	*0.719*	0.633	0.477	0.79
			*pSVGel*	0.668	*0.171*	*0.583*	*0.753*	0.668	0.518	0.818
			*pSVGmag*	0.514	*0.167*	*0.431*	*0.597*	0.514	0.346	0.683
			*aQRST*	0.681	*0.170*	*0.596*	*0.766*	0.681	0.543	0.819
			*aSVGaz*	0.55	*0.170*	*0.465*	*0.635*	0.55	0.396	0.704
			*aSVGel*	0.652	*0.171*	*0.566*	*0.738*	0.652	0.531	0.774
			*aSVGmag*	0.55	*0.170*	*0.465*	*0.635*	0.55	0.373	0.726
			*SAIQRST*	0.661	*0.171*	*0.576*	*0.746*	0.661	0.515	0.807
*2 years*	*84*	*15,632*	*HR*	0.618	*0.129*	*0.554*	*0.682*	0.618	0.522	0.715
			*QTc*	0.625	*0.129*	*0.561*	*0.689*	0.625	0.528	0.722
			*QRS*	0.682	*0.127*	*0.618*	*0.746*	0.682	0.595	0.769
			*pQRST*	0.738	*0.123*	*0.677*	*0.799*	0.738	0.661	0.814
			*pSVGaz*	0.673	*0.127*	*0.609*	*0.737*	0.673	0.567	0.779
			*pSVGel*	0.64	*0.128*	*0.576*	*0.704*	0.64	0.549	0.731
			*pSVGmag*	0.503	*0.124*	*0.441*	*0.565*	0.503	0.399	0.608
			*aQRST*	0.689	*0.127*	*0.626*	*0.752*	0.689	0.602	0.776
			*aSVGaz*	0.611	*0.129*	*0.547*	*0.675*	0.611	0.504	0.717
			*aSVGel*	0.608	*0.129*	*0.544*	*0.672*	0.608	0.515	0.701
			*aSVGmag*	0.545	*0.127*	*0.482*	*0.608*	0.545	0.448	0.642
			*SAIQRST*	0.687	*0.127*	*0.624*	*0.750*	0.687	0.600	0.775
*5 years*	*495*	*15,221*	*HR*	0.581	*0.053*	*0.554*	*0.608*	0.581	0.508	0.654
			*QTc*	0.604	*0.054*	*0.577*	*0.631*	0.604	0.548	0.659
			*QRS*	0.681	*0.053*	*0.655*	*0.717*	0.681	0.62	0.743
			*pQRST*	0.715	*0.052*	*0.689*	*0.741*	0.715	0.652	0.779
			*pSVGaz*	0.646	*0.053*	*0.619*	*0.673*	0.646	0.572	0.72
			*pSVGel*	0.624	*0.054*	*0.597*	*0.651*	0.624	0.577	0.671
			*pSVGmag*	0.486	*0.051*	*0.460*	*0.512*	0.486	0.403	0.568
			*aQRST*	0.691	*0.053*	*0.665*	*0.717*	0.691	0.633	0.749
			*aSVGaz*	0.622	*0.054*	*0.595*	*0.649*	0.622	0.557	0.687
			*aSVGel*	0.612	*0.054*	*0.585*	*0.639*	0.612	0.549	0.675
			*aSVGmag*	0.512	*0.052*	*0.486*	*0.538*	0.512	0.444	0.581
			*SAIQRST*	0.657	*0.053*	*0.630*	*0.684*	0.657	0.594	0.72
*> 5 years*	*320*	*15,396*	*HR*	0.55	*0.066*	*0.517*	*0.583*	0.55	0.507	0.593
			*QTc*	0.577	*0.066*	*0.544*	*0.610*	0.577	0.532	0.622
			*QRS*	0.655	*0.066*	*0.622*	*0.688*	0.655	0.617	0.693
			*pQRST*	0.71	*0.064*	*0.678*	*0.742*	0.71	0.668	0.753
			*pSVGaz*	0.612	*0.066*	*0.579*	*0.645*	0.612	0.567	0.657
			*pSVGel*	0.602	*0.066*	*0.567*	*0.633*	0.602	0.561	0.643
			*pSVGmag*	0.491	*0.064*	*0.459*	*0.523*	0.491	0.444	0.537
			*aQRST*	0.675	*0.066*	*0.642*	*0.708*	0.675	0.636	0.714
			*aSVGaz*	0.587	*0.066*	*0.554*	*0.610*	0.587	0.544	0.629
			*aSVGel*	0.596	*0.066*	*0.563*	*0.629*	0.596	0.555	0.636
			*aSVGmag*	0.523	*0.065*	*0.491*	*0.555*	0.523	0.479	0.568
			*SAIQRST*	0.635	*0.066*	*0.602*	*0.668*	0.635	0.598	0.672

**Table 3:** Integrated Discrimination Improvement (IDI) and Net Reclassification Improvement (NRI) for ECG metrics added to clinical predictors of SCD and nonSCD outcomes (age, sex, race, coronary heart disease, stroke, hypertension, diabetes)

Outcome	Prediction model	Reclassification index	1–90 days (high risk ≥25%)	91–180 d (high risk ≥25%)	181–365 d (high risk ≥25%)	365–730 d (high risk ≥10%)	731–1825 d (high risk ≥10%)	> 5 years (high risk ≥10%)
Sudden cardiac death	Clinical + QTc, ms	Absolute IDI (*P*-value)	0.063(0.109)	0.007(0.437)	0.001(0.695)	0.004(0.236)	0.001(0.020)	0.0003(0.424)
		Event Reclassified Up	2/11(18%)	0	2/47(4.3%)	3/84(3.6)	11/495(2%)	0
		Category-free NRI (*P*-value)	0.331(0.106)	−0.049(0.473)	0.057(0.114)	0.011(0.677)	0.005(0.226)	−0.003(0.432)
	Clinical + QRS, ms	Absolute IDI (*P*-value)	0.012(0.430)	0.004(0.785)	0.013(0.097)	0.003(0.215)	0.002(0.00006)	0.001(0.070)
		Event Reclassified Up	0	0	0	3/84(3.6%)	19/495(3.8%)	1/320(0.3%)
		Category-free NRI (*P*-value)	−0.023(0.839)	− 0.015(0.853)	0.058(0.018)	0.003(0.919)	0.009(0.032)	−0.009(0.198)
	Clinical + heart rate, bpm	Absolute IDI (P-value)	0.00004(0.978)	0.026(0.244)	0.020(0.030)	0.0003(0.664)	−0.0002(0.402)	0.0005(0.410)
		Event Reclassified Up	0	0	4/47(8.5%)	3/84(3.6%)	11/495(2.2%)	3/320(1%)
		Category-free NRI (*P*-value)	0(1.0)	−0.067(0.420)	0.029(0.671)	0.034(0.109)	0.003(0.433)	−0.004(0.630)
	Clinical+ Area QRS-T angle, °	Absolute IDI (*P*-value)	0.001(0.784)	0.066(0.046)	0.0001(0.852)	0.005(0.195)	0.005(< 0.00001)	0.002(0.029)
		Event Reclassified Up	0	1/16(6.3%)	0	2/84(2.4%)	46/495(9.6%)	1/320(0.3%)
		Category-free NRI (*P*-value)	0(1.0)	−0.032(0.801)	−0.006(0.317)	0.035(0.118)	0.021(0.003)	−0.006(0.308)
	Clinical+ Peak QRS-T angle, °	Absolute IDI (*P*-value)	0.032(0.328)	0.048(0.083)	−0.00001(0.986)	0.007(0.084)	0.007(< 0.00001)	0.001(0.045)
		Event Reclassified Up	2/11(18%)	1/16(6.3%)	0	5/84(6.0)	52/495(10.5%)	1/320(0.3%)
		Category-free NRI (*P*-value)	0.216(0.246)	−0.101(0.420)	−0.012(0.157)	0.041(0.252)	0.021(0.006)	−0.015(0.067)
	Clinical+ Area SVG azimuth, °	Absolute IDI (*P*-value)	0.012(0.607)	0.007(0.651)	0.002(0.665)	0.020(0.001)	0.002(0.004)	−0.0002(0.0008)
		Event Reclassified Up	2/11(18%)	0	1/47(2.1%)	7/84(7.1%)	21/495(4.2%)	0
		Category-free NRI (*P*-value)	0.277(0.068)	−0.050(0.541)	0.023(0.398)	0.055(0.152)	0.003(0.578)	0.0002(0.083)
	Clinical+ Peak SVG azimuth, °	Absolute IDI (*P*-value)	0.015(0.608)	0.045(0.141)	−0.0001(0.880)	0.017(0.005)	0.003(0.00005)	−0.0001(0.568)
		Event Reclassified Up	2/11(18%)	0	0	5/84(6%)	27/495(5.5%)	0
		Category-free NRI (P-value)	0.277(0.068)	−0.133(0.221)	−0.006(0.564)	0.020(0.598)	0.004(0.538)	0.0009(0.003)
	Clinical + Area SVG elevation, °	Absolute IDI (*P*-value)	0.002(0.735)	0.050(0.063)	0.004(0.398)	−0.00001(0.990)	0.0001(0.576)	−0.0004(0.045)
		Event Reclassified Up	0	1/16(6.3%)	0	3/84(3.6%)	11/495(2.2%)	0
		Category-free NRI (*P*-value)	0.038(0.317)	−0.118(0.349)	0.018(0.257)	0.037(0.093)	0.001(0.847)	0.0004(0.034)
	Clinical + Peak SVG elevation, °	Absolute IDI (*P*-value)	0.003(0.663)	0.046(0.112)	0.002(0.463)	0.0004(0.879)	0.0002(0.387)	−0.0003(0.023)
		Event Reclassified Up	0	0	0	5/84(6%)	7/495(1.4%)	0
		Category-free NRI (*P*-value)	0.038(0.317)	−0.084(0.276)	0.006(0.564)	0.070(0.017)	−0.004(0.240)	0.0005(0.020)
	Clinical + SAI QRST,mV*ms	Absolute IDI (*P*-value)	0.0001(0.755)	0.023(0.339)	0.002(0.522)	0.012(0.040)	0.002(0.0004)	0.001(0.061)
		Event Reclassified Up	0	0	0	1/84(1.2%)	23/495(4.6%)	1/320(0.3%)
		Category-free NRI (*P*-value)	0(1.0)	−0.151(0.151)	0.006(0.564)	0.007(0.778)	0.013(0.005)	0.0005(0.909)
	Clinical + SVG magnitude, μV	Absolute IDI (*P*-value)	0.015(0.566)	−0.0002(0.954)	0.0002(0.884)	0.0009(0.188)	−0.0002(0.00003)	0.00007(0.764)
		Event Reclassified Up	0	0	0	2/84(2.4%)	0	0
		Category-free NRI (*P*-value)	−0.023(0.839)	− 0.052(0.083)	− 0.012(0.157)	0.018(0.318)	0.0001(0.371)	− 0.002(0.516)
	Clinical + Peak SVG mag, μV	Absolute IDI (*P*-value)	0.001(0.748)	0.0003(0.959)	0.0002(0.869)	0.0006(0.095)	−0.00006(0.676)	−0.0002(0.189)
		Event Reclassified Up	0	0	0	3/84(3.6%)	7/495(1.4%)	0
		Category-free NRI (*P*-value)	0(1.0)	−0.052(0.180)	− 0.012(0.157)	0.032(0.135)	0.004(0.144)	0.0003(0.102)
Non-Sudden cardiac death	Clinical + QTc, ms	Absolute IDI (P-value)	0.069(0.170)	0.006(0.667)	0.015(0.200)	0.0001(0.843)	0.003(< 0.00001	0.001(0.040)
		Event Reclassified Up	0	0	2/47(2.3%)	0	46/711(6.5%)	10/394(2.5%)
		Category-free NRI (*P*-value)	0.200(0.059)	0(1.0)	−0.006(0.880)	−0.011(0.225)	0.002(0.749)	0.016(0.064)
	Clinical + QRS, ms	Absolute IDI (*P*-value)	0.028(0.467)	0.187(0.001)	−0.002(0.702)	0.0007(0.623)	0.002(0.0001)	0.001(0.051)
		Event Reclassified Up	0	2/16(12.5%)	0	0	34/711(4.8%)	10/394(2.5%)
		Category-free NRI (*P*-value)	−0.011(0.919)	0.136(0.230)	0.014(0.561)	0.011(0.225)	0.004(0.395)	0.009(0.337)
	Clinical + heart rate, bpm	Absolute IDI (*P*-value)	0.060(0.160)	−0.0001(0.981)	0.0005(0.909)	0.0003(0.779)	0.005(< 0.00001)	0.002(0.010)
		Event Reclassified Up	0	0	1/47(2.1%)	0	89/711(12.5%)	14/394(3.6%)
		Category-free NRI (*P*-value)	0.200(0.059)	0(1.0)	0.037(0.158)	−0.0002(0.990)	0.014(0.060)	0.014(0.207)
	Clinical+ Area QRS-T angle, °	Absolute IDI (*P*-value)	0.053(0.272)	0.012(0.625)	0.059(0.002)	0.007(0.120)	0.010(< 0.00001)	0.004(0.0004)
		Event Reclassified Up	1/11(9%)	0	4/47(8.5%)	0	108/711(15%)	19/394(4.8%)
		Category-free NRI (*P*-value)	0.120(0.411)	−0.049(0.499)	0.060(0.286)	0.084(0.0003)	0.017(0.034)	0.021(0.097)
	Clinical+ Peak QRS-T angle, °	Absolute IDI (*P*-value)	0.169(0.026)	0.077(0.030)	0.072(0.0004)	0.005(0.180)	0.009(< 0.00001)	0.005(0.00002)
		Event Reclassified Up	1/11(9%)	0	6/47(12.8%)	0	114/711(16%)	24/394(6.1%)
		Category-free NRI (*P*-value)	0.120(0.411)	−0.084(0.276)	0.01(0.167)	0.052(0.006)	0.017(0.033)	0.034(0.011)
	Clinical+ Area SVG azimuth, °	Absolute IDI (*P*-value)	0.183(0.003)	0.123(0.010)	0.014(0.093)	0.0008(0.534)	0.003(< 0.00001)	0.003(0.0006)
		Event Reclassified Up	1/11(9%)	0	1/47(2.1%)	0	57/711(8.0%)	13/394(3.3%)
		Category-free NRI (*P*-value)	0.200(0.202)	−0.049(0.574)	−0.005(0.899)	− 0.0002(0.994)	0.009(0.109)	0.06(0.112)
	Clinical+ Peak SVG azimuth, °	Absolute IDI (*P*-value)	0.164(0.015)	0.005(0.740)	0.007(0.315)	0.0003(0.642)	0.003(< 0.00001)	0.003(0.001)
		Event Reclassified Up	1/11(9%)	0	2/47(2.3%)	0	45/711(6.3%)	17/394(4.3%)
		Category-free NRI (P-value)	0.240(0.138)	0.017(0.317)	−0.008(0.840)	−0.002(0.782)	0.003(0.600)	0.027(0.019)
	Clinical + Area SVG elevation, °	Absolute IDI (P-value)	0.025(0.398)	0.058(0.057)	0.007(0.370)	0.0008(0.356)	0.0005(0.015)	0.0006(0.044)
		Event Reclassified Up	0	0	4/47(8.5%)	0	16/711(2.3%)	9/394(2.3%)
		Category-free NRI (*P*-value)	−0.051(0.608)	0(1.0)	0.114(0.007)	−0.007(0.549)	− 0.005(0.216)	0.018(0.025)
	Clinical + Peak SVG elevation, °	Absolute IDI (*P*-value)	0.039(0.273)	0.017(0.193)	0.018(0.081)	0.0005(0.703)	0.002(0.00007)	0.0004(0.081)
		Event Reclassified Up	1/11(9%)	0	4/47(8.5%)	0	34/711(4.8%)	4/394(1.0%)
		Category-free NRI (P-value)	0.189(0.319)	0.017(0.317)	0.068(0.210)	0.014(0.221)	−0.002(0.743)	0.009(0.065)
	Clinical + SAI QRST,mV*ms	Absolute IDI (P-value)	0.040(0.422)	0.018(0.528)	0.027(0.059)	0.019(0.022)	0.004(< 0.00001)	0.0006(0.093)
		Event Reclassified Up	0	0	4/47(8.5%)	1/84(1%)	49/711(6.9%)	3/394(0.8%)
		Category-free NRI (P-value)	0.109(0.392)	−0.084(0.251)	0.054(0.290)	0.098(0.003)	0.002(0.770)	0.003(0.580)
	Clinical + SVG magnitude, μV	Absolute IDI (P-value)	0.028(0.332)	−0.002(0.886)	−0.002(0.480)	0.026(0.004)	0.0008(0.00008)	0.0002(0.00008)
		Event Reclassified Up	0	0	0	1/84(1%)	10/711(1.4%)	0
		Category-free NRI (P-value)	0.029(0.799)	0(1.0)	0.006(0.317)	0.090(0.021)	−0.004(0.178)	−0.0004(0.059)
	Clinical + Peak SVG mag, μV	Absolute IDI (P-value)	0.027(0.355)	0.020(0.433)	−0.0004(0.907)	0.016(0.032)	0.0002(< 0.00001)	0.0003(0.067)
		Event Reclassified Up	0	0	0	1/84(1%)	2/711(0.3%)	3/394(0.8%)
		Category-free NRI (P-value)	0.029(0.799)	−0.067(0.348)	0(1.0)	0.098(0.005)	−0.001(0.473)	0.004(0.482)

### Comparison of area-based versus peak-based SVG and QRS-T angle measurements

**Fig. 7:**
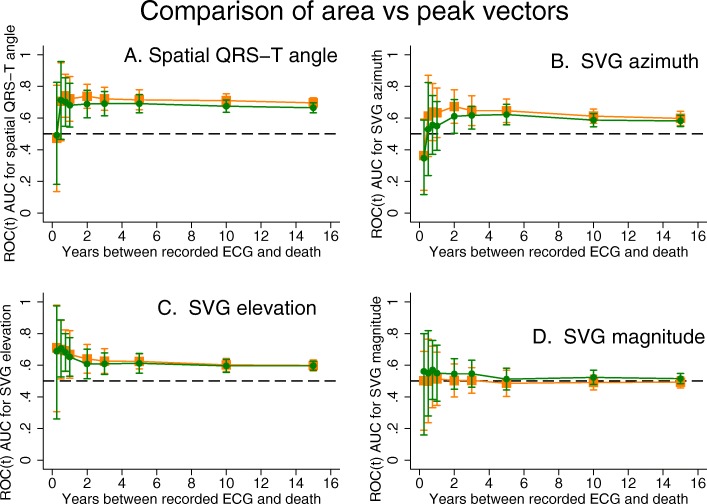
Comparison of time-dependent AUC (with 95% CI) for windows of SCD prediction 3, 6, 9 months, 1, 2, 3, 5, 10, 15 years, for peak-based SVG vector measurements (orange squares) vs. area-based SVG vector measurements (green circles): **a** QRS-T angle, **b** SVG azimuth, **c** SVG elevation, **d** SVG magnitude

SCD predictive accuracy of peak-vector-based versus area-based spatial QRS-T angle, SVG direction, and SVG magnitude was similar (Fig. 7), with a trend towards slightly better prediction by peak-vector-based QRS-T angle and SVG azimuth, as compared to area-based QRS-T angle and SVG azimuth metrics.

#### Internal validation

Results of internal validation are shown in Additional file 1: Table S1 and Figs. 2, 3, 4. As expected, long-term prediction of SCD was consistent across all 5 study partitions, whereas short-term (within 3–9 months) prediction was unreliable for most ECG variables (except SVG elevation). Consistently, statistical power was reliably sufficient for ROC(t) AUC analyses for outcomes occurring at least 1 year after ECG recording, and beyond (Table 2).

## Discussion

In this study, we described the dynamic predictive accuracy of ECG and VCG biomarkers of two competing mortality outcomes: SCD and non-SCD within a survival framework. Within the identified dynamic predictors of SCD, there was a distinction between markers predicting short-term events (within 6 months) and markers predicting more intermediate- and long-term events. This may represent the difference between markers heralding SCD (triggers or transient substrates) versus markers identifying persistent substrate. As expected, transient substrate of non-SCD (describing structural heart disease substrate) was characterized by wide QRS-T angle, SVG vector pointing backward (towards LV), wide QRS, prolonged QTc, and increased heart rate. A transient substrate of SCD was characterized by SVG vector pointing upward (towards the outflow tract). Dynamic predictive accuracy of ECG and VCG biomarkers of SCD should be taken into account for development of dynamic and life-long prediction of SCD and non-SCD. Importantly, the addition of GEH metrics (but not QTc) to known demographic and clinical risk factors (age, sex race, CHD, stroke, diabetes, and hypertension) significantly improved reclassification of risk, supporting inclusion of GEH metrics into dynamic risk scores for SCD.

### Triggers, or transient substrate of SCD event

An SCD event represents a “perfect storm,” requiring both susceptible anatomical/functional substrate and a trigger/transient initiating event [21]. Short-term predictors of SCD in our study reflect possible SCD triggers. The SVG vector direction predicting short-term SCD risk differed from SVG vector direction of the intermediate- and long-term risk of SCD. The short-term risk was uniquely predicted by an SVG vector pointing upward (toward the outflow tract), suggesting that short total recovery time in the outflow tracts (as the SVG vector points towards an area with the shortest total refractory time) [22] may represent an SCD trigger. SVG azimuth was the only ECG metric which improved reclassification of the risk beyond known clinical and demographic risk factors. Indeed, it is known that “malignant” idiopathic ventricular fibrillation and polymorphic ventricular tachycardia can be triggered by ventricular ectopy arising from the outflow tracts [23]. Early cardiac development affects the generation of electrophysiological heterogeneities in the adult heart [24]. There may be a genetic basis for this phenomenon as GEH-associated genetic polymorphisms indicated the involvement of *HAND1* and *TBX3* genes [5], both of which play a role in outflow tract development.

### Intermediate and long-term substrates of SCD

All ECG measurements (except SVG magnitude) predicted SCD long-term. The long-term substrate of SCD was characterized by an SVG vector pointing backward (toward the LV), a wide spatial QRS-T angle, and a large SAI QRST. Reliable long-term prediction of SCD offers an opportunity for early preventive intervention. Many GEH-associated genetic loci are implicated in cardiac development [5]. Further studies of the underlying biology behind GEH-associated loci will help to uncover novel mechanisms of SCD and develop primary prevention strategies. A recent case-control genome-wide association study of sudden cardiac arrest [25] did not identify any variants at genome-wide statistical significance. An ideal case-control study of paroxysmal life-threatening arrhythmias (e.g. SCD) would require evidence of freedom from arrhythmogenic substrate in controls, which is difficult to achieve. As both trigger and substrate are required for the development of sudden cardiac arrest, a low yield from a case-control genome-wide association study of sudden cardiac arrest is to be expected. In contrast, genomic studies of electrophysiological substrates have the advantage of a more accurate measurement of phenotype and larger statistical power (as an outcome is a continuous variable), providing higher yield.

### Dynamic predictive accuracy of biomarkers within a survival framework

The dynamic nature of SCD risk is well-recognized. However, the dynamic predictive accuracy of SCD risk markers has not been previously studied. Our large prospective epidemiological study used repeated ECG measures, obtained at five follow-up visits, which ensured stable estimates of the dynamic predictive accuracy of ECG biomarkers within a survival framework. An analytical framework for the assessment of a dynamic predictive accuracy of biomarkers for censored survival data was developed fairly recently [17]. Heagerty et al. [17] showed that a simple estimator based on Kaplan-Meier method has serious shortcomings for characterization of accuracy for censored survival outcomes, and developed the nearest neighbor estimator as a valid ROC solution for prediction accuracy assessment, allowing the censoring process to depend on the marker. In this study, we used an analytical approach to answer an agnostic predictive accuracy question. To mimic the real-life clinical scenarios, we intentionally did not adjust for confounders and therefore did not comment on the independence of association of ECG biomarkers with SCD at any given time point. There were noticeable differences in the clinical characteristics of study participants who died suddenly within 3 months after ECG recording, as compared to those who experienced SCD 5 years after ECG recording. Nevertheless, observed dissimilarities in a dynamic predictive accuracy of ECG biomarkers suggested different mechanisms behind short-term SCD triggers (or transient substrates) and long-term SCD substrates. A study of SCD triggers is objectively difficult to conduct. The methodological approach of the dynamic predictive accuracy of ECG biomarkers within the survival framework can provide unique perspective on transient substrates and triggers of SCD, which prompts further investigation.

### Global electrical heterogeneity

The GEH concept is based on Wilson’s ventricular gradient [22, 26]. SVG defines a vector that characterizes the magnitude and direction of the steepest gradient between the longest and the shortest total recovery time across the entire heart, both left and right ventricles [27]. SVG vector is directed towards a zone with the shortest total recovery time. Therefore, GEH is a global measure of the dispersion of total recovery time across the heart, a marker of an underlying arrhythmogenic substrate, encompassing dispersion in both activation and refractoriness. We comprehensively characterize GEH by measuring five features of the SVG vector (Fig. 1): SVG magnitude, SVG direction (azimuth and elevation), QRS-T angle, and SVG’s scalar SAI QRST. The spatial QRS-T angle [28] is a well-known marker of the risk of SCD and cardiovascular mortality. SAI QRST is a scalar analog of the SVG [15, 29–31], associated with ventricular tachyarrhythmias in heart failure [29, 30, 32]. We previously showed that GEH is associated with SCD after adjustment for traditional ECG metrics and a comprehensive list of cardiovascular risk factors [4]. Five GEH metrics only weekly correlate with each other and traditional ECG measures, [5] and, therefore, are well-suited for inclusion in SCD risk scores. Importantly, GEH genome-wide association study [5] provided first clues about underlying biology behind GEH, which can lead to the future development of novel methods of SCD prevention. In vectorcardiography, there are two major approaches to define spatial vectors: either measuring spatial peak or area vectors [28]. In our study, some peak-based GEH metrics outperformed area-based GEH metrics. This finding may be at least partially explained by the fact that we used a physiologically sound definition of the heart vector origin point and time-coherent global median beat [14], which permitted accurate measurement of peak vectors. GEH metrics are highly reproducible [33], which is an essential factor for their future implementation in clinical practice. Of note, GEH can be calculated using 12-lead ECG that was recorded by any manufacturer ECG machine. The open-source software code for GEH calculation is provided at https://github.com/Tereshchenkolab/Origin and https://www.physionet.org/content/geh.

### Clinical implications

Our study is the first step towards the development of dynamic SCD risk prediction. Recently, we developed an SCD risk score [4], available at www.ecgpredictscd.org. However, our SCD risk model, similarly to other risk models [34, 35], includes risk factors that were measured once at baseline. Further improvement of SCD risk stratification requires knowledge of the dynamic predictive accuracy of ECG biomarkers of SCD. After validation of our study findings in another cohort, recommendations regarding an optimal frequency of ECG recordings can be developed.

### Strengths and limitations

The strength of our study derives from the large prospective cohort design, with five longitudinal ECG recordings, long-term (median 24 years) follow-up, and a well-adjudicated SCD outcome. However, limitations of the study should be taken into account. The small number of events within 3 and 6 months after ECG recording limited the statistical power of SCD trigger analyses. It is worth noting that 9 out of 11 ARIC participants who succumbed to SCD within 3 months after ECG recording were men. We cannot rule out the possibility that the observed transient substrate of SCD is sex-specific. Further studies of SCD triggers in women are needed. While we performed robust internal validation of our findings using bootstrapping in five partitions of the dataset, replication of the SCD trigger analyses in another prospective cohort is needed. Nevertheless, this is the largest prospective study of SCD triggers and substrates, suggesting differences between long-term and transient SCD-specific and non-SCD-specific substrates [36]. In this study, correlation between GEH metrics and heart rate was weak (r values between 0.1–0.2), and we did not normalize GEH metrics by heart rate. However, further studies are needed to determine whether normalization by heart rate can further improve the predictive value of GEH.

## Conclusions

Dynamic predictive accuracy of ECG and VCG biomarkers should be taken into account for the development of dynamic risk scores of competing SCD risk. The distinction between markers predicting short-term and long-term events may represent the difference between markers heralding SCD (triggers or transient substrates) versus markers identifying persistent substrate.

## Supplementary information

**Additional file 1: Table S1**. Time-dependent AUC with 95% Confidence Interval for prediction of SCD and non-SCD, for windows of prediction 3, 6, 9 months, 1, 2, 3, 5, 10, 15 years for traditional ECG markers, and GEH-ECG in five partitions of the study dataset. See separate excel file. _lb = lower bound; _ub = upper bound. HR = heart rate; qtc = QTc interval; qrs = QRS interval; pQRST = peak QRS-T angle; aQRST = area QRS-T angle; pSVGaz = peak SVG azimuth; aSVGaz = area SVG azimuth; pSVGel = peak SVG elevation; aSVGel = area SVG elevation; pSVGmag = peak SVG magnitude; aSVGmag = area SVG magnitude. SAI = Sum Absolute QRST Integral (SAI QRST).

## Data Availability

After the ethical approval at a requestor institution, the de-identified ARIC data can be obtained from the National Heart, Lung, and Blood Institute – maintained BioLINCC repository [37], and via ARIC Coordinating Center at the University of North Carolina—Chapel Hill [38]. The BioLINCC website [37] includes detailed information about the process to obtain such data. Details about the procedures for data request from the ARIC Coordinating Center at the University of North Carolina—Chapel Hill can be found online [38]. GEH results were reported to the ARIC Coordinating Center by the ARIC ancillary study “Novel ECG measures and risk of sudden cardiac death” (principal investigator Tereshchenko). We provided open-source MATLAB (MathWorks, Natick, MA, USA) code at: https://github.com/Tereshchenkolab/Global-Electrical-Heterogeneity, and https://github.com/Tereshchenkolab/Origin. SCD risk calculator is available at http://www.ecgpredictscd.org/.

## Abbreviations

AF: Atrial fibrillation
ARIC: Atherosclerosis risk in community study
AUC: Area under the curve
AV: Atrioventricular
CHD: Coronary heart disease
CI: Confidence interval
CVD: Cardiovascular disease
ECG: Electrocardiogram
GEH: Global electrical heterogeneity
HF: Heart failure
IDI: Integrated discrimination improvement
LV: Left ventricle
MI: Myocardial infarction
Non-SCD: Non-sudden cardiac death
NRI: Net reclassification improvement
PAD: Peripheral artery disease
ROC: Receiver operating characteristic
RVOT: Right ventricular outflow tract
SAI QRST: Sum absolute QRST integral
SCD: Sudden cardiac death
SD: Standard deviation
SVG: Spatial ventricular gradient
US: United States
VM: Vector magnitude
